# Focal Retrograde Amnesia: Voxel-Based Morphometry Findings in a Case without MRI Lesions

**DOI:** 10.1371/journal.pone.0026538

**Published:** 2011-10-19

**Authors:** Bernhard Sehm, Stefan Frisch, Angelika Thöne-Otto, Annette Horstmann, Arno Villringer, Hellmuth Obrig

**Affiliations:** 1 Clinic of Cognitive Neurology, University of Leipzig, Leipzig, Germany; 2 Department for Neurology, Max-Planck-Institute for Human Cognitive and Brain Sciences, Leipzig, Germany; University of Leuven, Belgium

## Abstract

Focal retrograde amnesia (FRA) is a rare neurocognitive disorder presenting with an isolated loss of retrograde memory. In the absence of detectable brain lesions, a differentiation of FRA from psychogenic causes is difficult. Here we report a case study of persisting FRA after an epileptic seizure. A thorough neuropsychological assessment confirmed severe retrograde memory deficits while anterograde memory abilities were completely normal. Neurological and psychiatric examination were unremarkable and high-resolution MRI showed no neuroradiologically apparent lesion. However, voxel-based morphometry (VBM)-comparing the MRI to an education-, age-and sex-matched control group (n = 20) disclosed distinct gray matter decreases in left temporopolar cortex and a region between right posterior parahippocampal and lingual cortex. Although the results of VBM-based comparisons between a single case and a healthy control group are generally susceptible to differences unrelated to the specific symptoms of the case, we believe that our data suggest a causal role of the cortical areas detected since the retrograde memory deficit is the preeminent neuropsychological difference between patient and controls. This was paralleled by grey matter differences in central nodes of the retrograde memory network. We therefore suggest that these subtle alterations represent structural correlates of the focal retrograde amnesia in our patient. Beyond the implications for the diagnosis and etiology of FRA, our results advocate the use of VBM in conditions that do not show abnormalities in clinical radiological assessment, but show distinct neuropsychological deficits.

## Introduction

The loss of retrograde memory contrasted with normal learning of new information constitutes a rare memory disorder termed “focal retrograde amnesia” (FRA). Case studies demonstrated this syndrome to be associated with various neurological disorders such as traumatic brain injury, encephalitis, hypoxia or epilepsy [1]–[3]. Size and localization of related lesions vary and include neocortical, limbic and brain stem structures [1]–[3]. However, based on scientific evidence medial temporal, temporopolar and frontal cortices play a key role in remote memory functions and should thus be involved in the pathophysiology of FRA [4].

In patients without detectable brain lesions, the differentiation of a psychogenic cause is difficult. Here, the term “functional retrograde amnesia” has been coined [5], [6], leaving it controversial whether the syndrome is of purely psychogenic nature or whether subtle structural/metabolic changes may account for the memory impairment [5], [7]. Merging both concepts, Kopelman proposed that both organic and functional/psychogenic factors interactively contribute in the presentation of the deficit [8].

Here we report a case of persistent FRA after an epileptic seizure. Neurological examination was unremarkable and high-resolution MRI showed no neuroradiologically apparent lesion. However, voxel-based morphometry (VBM)-comparing the patient's MRI to a control group of male subjects of the same age and educational background-disclosed distinct gray matter decreases in left temporopolar and right posterior parahippocampal/lingual cortices.

## Methods

### Case report

The study adhered to the declaration of Helsinki and was approved by the ethics committee of the University of Leipzig. Both patient and healthy control subjects gave informed written consent to the participation in the study and the publication of this report.

In February 2009 a 24-year old male engineering student suffered from a sudden unwitnessed loss of consciousness. A prolonged postictal state and bitemporal sharp-wave-complexes in the initial EEG supported the diagnosis of an epileptic seizure. All other paraclinical measures (including CSF) were within normal limits. Under Lamotrigine therapy, follow-up standard EEGs and EEG-videomonitoring did not reveal any epileptic activity. Clinically the patient reported frequent déjà-vues (4 times per week) prior to the start of the effective anticonvulsant therapy.

Immediately after the seizure, the patient experienced a profound retrograde amnesia covering his entire prior life, while anterograde memory functions were unaffected. Initially this persisting FRA was considered dissociative in origin. Six months after the event the patient was admitted to our clinic. The patient and his parents reported that at first the amnestic symptoms were severe. The patient only recognized his parents but not his fiancée. He had been disoriented with regard to time, space and personal identity. Since then, symptoms had improved, but he still had extensive memory gaps regarding personal memories of e.g. places and persons extending back to his childhood. On neurological and psychiatric examination the right-handed patient was completely unremarkable.

### Neuropsychological testing

Formal neuropsychological testing covered both anterograde and retrograde memory functions. The following tests were deployed (please see also Table 1): California Verbal Learning Test (CVLT) [9], Wechsler Memory Scale revised (WMS-R) [10], Visueller und Verbaler Merkfähigkeitstest (VVM) [11], Wechsler Adult Intelligence Scale (WAIS) [12], Autobiographical Memory Interview (AMI) [13], Autobiographisches Altgedächtnisinterview (AAI) [14]. Additionally, for the assessment of general semantic memory we created a new questionnaire of 23 public events in analogy to an established but outdated questionnaire [15].

**Table 1:** Anterograde and retrograde memory test scores.

function	test	raw score (total)	percentile rank
*anterograde memory*			
verbal learning/delayed recall	CVLT total learning score/delayed recall	76(80)/15(16)	98/84
verbal encoding/delayed recall (30 min)	WMS-R logical memory I/II	39(40)/25(40)	91/36
visual encoding/delayed recall (30 min)	WMS-R visual reproduction I/II	38(40)/36(40)	78/29
verbal encoding/delayed recall (24 h)	VVM verbal I/II/forgetting rate	14(21)/12(21)/14%	31/29/43
visual encoding/delayed recall (24 h)	VVM visual I/II/forgetting rate	27(31)/29(31)/7%	72/80/88

*retrograde memory*			
general semantic memory	WAIS Vocabulary	56(66)	84
	WAIS Information	21(28)	>50
	WAIS Comprehension	26(35)	>50
	questionnaire on public events (years 2000–2006)*	18(23) $	
personal semantic memory	AMI Before school (up to 5 years)	4(5) *	<16
	AMI First school (5–11 years)	4(8) *	<16
	AMI Main School (11–18 years)	6,5(8) *	<16
	AAI (last 5 years)	**19(34)**	*below cutoff* §
episodic memory	AMI Before school (up to 5 years)	**1(3)**	<2
	AMI First school (5–11 years)	**0(3)**	<2
	AMI Main School (11–18 years)	**0(3)**	<2

Abnormal results (i.e. below a percentile rank of 2) are displayed in **bold**. For abbreviations please see methods section of the article.

*Percentiles below 16% are considered ‘borderline’.

§ This test is not normed according to percentile ranks but authors defined a cutoff , which indicates pathological results.

$ A new questionnaire of 23 public events was created. Comparison of the patient's performance to that of an age-matched male control group (n = 20; mean 24 years, range 22–26 years) revealed no significant difference using a modified t-test procedure [33].

Furthermore we performed tests for attention, executive function/intelligence, mood and psychiatric symptoms [16]–[22]. Please see Table 2 for a detailed listing of the applied tests.

**Table 2:** Test scores for attention, executive functions and mood/psychiatric symptoms.

function	test	raw score	percentile rank
*attention*			
alertness/speed of processing	TAP [16] alertness tonic/phasic	281 ms*/290 ms *	5/4
divided attention	TAP divided attention mean RT/misses	650 ms/0	18/>50
short term memory/working memory	WMS-R[10] digit span forward/backward	8/6.5	93/88

*intelligence/executive functions*			
logical reasoning	LPS-3[17]	35 (40)	96
STROOP (interference condition)	FWIT[18]	102 s	50
verbal fluency	RWT[19] phonological/semantic	26/**17**	50/1
figural fluency	H5PT-R[20]	45	84

*mood/psychiatric symptoms*			
	SCL-90-R[21]		
	GSI	0.47	79
	PSDI	1.27	66
	PST	33	79
	Somatization	4	69
	Obsessive-compulsive	8 *	84
	Interpersonal sensitivity	6	76
	Depression	9 *	86
	Anxiety	2	58
	Hostility	1	46
	Phobic Anxiety	7 *	97
	Paranoid Ideation	1	42
	Psychoticism	3	76
	BDI[22]	5	normal

The results of the neuropsychological testing are displayed as raw data (in brackets the total number of items in the specific test) and the percentile rank, if applicable. Abnormal results (i.e. below a percentile rank of 2) are displayed in **bold**.

*Percentiles below 16% (and >84% for the SCL-90) are considered ‘borderline’.

### Control subjects

20 male subjects (age 24.2±1.9 SD) served as controls for the VBM analysis. All subjects were healthy and had no history of any neurological or psychiatric disease, which was assessed by a certified neurologist. To attenuate the risk of accidental differences of memory unrelated cognitive abilities between the single patient and the control group, we carefully selected the controls with regard to the educational status. Therefore only age-and sex-matched volunteers, who had passed their final high-school exam (‘Abitur’) with comparable success and were currently university students of comparable status were included.

### Acquisition and analysis of structural MRI data

MRI data was acquired on 3 Tesla Magnetom Tim Trio scanner (Siemens, Erlangen, Germany) using a 32-channel head coil. T1-weighted images were acquired using a MPRAGE sequence (TR = 1.3 s; TE = 3.46 ms; flip angle = 10°; FOV = 256 mm×240 mm; 176 sagittal slices; voxel size = 1×1×1.5 mm).

Pre-processing of T1-weighted images was performed using SPM5 (Wellcome Trust Centre for Neuroimaging, UCL, London; UK; http://www.fil.ion.ucl.ac.uk/spm) implemented in the VBM Toolbox 5.1 (Christian Gaser, Department of Psychiatry, University of Jena, Germany; http://dbm.neuro.uni-jena.de/vbm.html) under MatLab 7.7 (The MathWorks Inc., Sherborn, MA, USA). Standard routines and default parameters of the VBM 5.1 toolbox were applied. Images were bias corrected, pre-registered to standardized Montreal Neurological Institute (MNI) space using rigid-body transformation (with translation and rotation only) and segmented using the “unified segmentation” approach [23]. Segmentation in SPM5 was based on a modified gaussian mixture model to avoid misclassification. Information was combined from the intensity distribution of the image and prior information for all tissue classes by using prior probability maps that were derived from a large number of subjects. The prior probability maps were warped to the data to minimize the impact of template and priors. To remove isolated voxels of one tissue class within a cluster of voxels belonging to a different tissue class, a hidden Markov random field model with adaptive weighting was used. The warping to MNI space was performed using both, linear and non-linear transformations. Spatial normalization expands and contracts some brain regions. Grey matter segments were therefore modulated (i.e., scaled) by the Jacobian determinants of the deformations to account for local expansion and compression introduced by non-linear transformation. Finally, the grey matter images were smoothed with an 8-mm full-width at half-maximum (FWHM) isotropic gaussian kernel. This was done to reduce errors related to intersubject variability in local anatomy and to render the imaging data more normally distributed.

For statistical analysis, voxel-wise gray values of the patient were compared to those of the control group. We assumed a t-distribution of the control group data and used a 2-sample t-test (group 1: patient, group 2: controls). A covariate accounted for subjects' age. Data were analyzed on the whole-brain level and were corrected for multiple comparisons using the Family Wise Error correction (FWE) with p<.01. Results were corrected for non-isotropic smoothness [24]. Stereotactic coordinates are reported in MNI space.

## Results

### Neuropsychological test results

The neuropsychological assessment revealed average to above-average anterograde, but defective retrograde memory. A pronounced degradation of the episodic memory was found throughout his premorbid life (Table 1 details the memory test results). Due to the patient's young age, a number of items of retrograde memory tests did not apply (e.g. wedding, children, former hospital visits). Thus the time period of 5 years before onset of the amnesia was not fully covered by the test. Therefore we additionally conducted a detailed interview, which confirmed severe episodic memory deficits. Personal semantic memory revealed borderline results, since relevant personal information had been reacquired. The patient was able to describe episodes of his life according to what parents and friends had told him, but did fail to provide details as are characteristic of personal memories. General semantic memory showed no abnormality. No clear temporal gradient of the amnestic syndrome was found.

Apart from the mnestic deficit, tests for verbal fluency and processing speed were below average. Otherwise cognitive testing was unremarkable (Table 2).

With regard to a potential psychodynamic cause of the memory deficit, repeated anamnestic exploration and clinical observation throughout several weeks of therapy did not reveal any plausible psychogenic cause. This was supported by interviews with his relatives. The patient showed symptoms of mood disturbance and was insecure in social interaction, both clearly reactions to his memory impairment. In order to avoid potentially embarrassing situations, when meeting people he had formerly known, he avoided social contact. He also was anxious about his university career. These symptoms corresponded with the results of a formal evaluation of psychopathological symptoms [21] (see Table 2). No symptoms of anxiety or depression had been apparent prior to the amnesia.

Neuropsychological therapy focused on helping him overcome social avoidance. Strategies were developed how to react when meeting people he could not remember. Regarding his work, mostly consisting of computer programming, he had largely preserved semantic and procedural knowledge. Information that he did not remember was easily relearnt with the help of his colleagues.

### Imaging results

**Figure 1:**
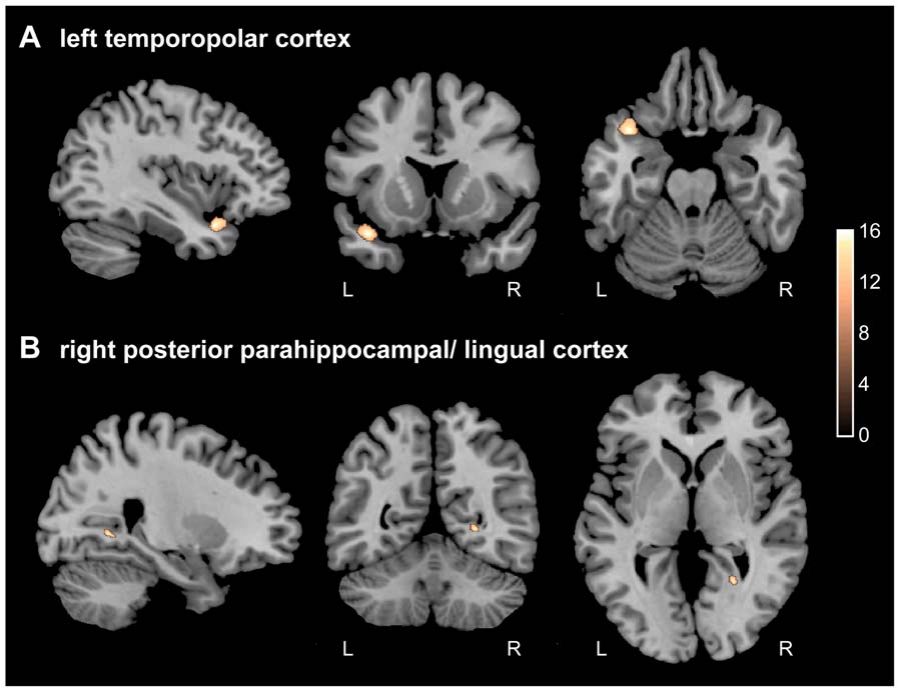
Gray matter decreases of the patient compared to healthy controls (n = 20). VBM analysis revealed regional decreases in gray matter density (patient vs. 20 age- and sex-matched controls) in 2 clusters. A) left temporopolar (cluster-size of local maximum 687, T = 16.30, Z = 6.54) and B) right posterior parahippocampal/lingual cortex (cluster-size of local maximum 700; T = 15.62; Z = 6.45). All values are FWE-corrected for multiple comparisons, p<0.01, color bar represents color of respective t-values.

High-resolution MRI showed no lesion or obvious morphological abnormality as confirmed by an experienced neuroradiologist. VBM was used to compare the patient's MRI to a group of 20 age-matched male control subjects (Figure 1). Here, the patient showed highly significant gray matter volume decreases within portions of the left temporopolar cortex (MNI coordinates: −39, 13 −22; cluster-size of local maximum 687; T = 16.30; Z = 6.54) and a region in the border of right posterior parahippocampal and lingual cortex (MNI coordinates: 25, −55, 1; cluster-size of local maximum 700; T = 15.62; Z = 6.45).

## Discussion

To our knowledge this is the first report on VBM-based demonstration of subtle changes in the memory network in a patient suffering from FRA without neuroradiologically detectable MRI-abnormalities. The aetiology of these changes remains intricate, but left temporopolar and right posterior parahippocampal/lingual gray matter decreases indicate a subtle abnormality in key areas of episodic memory and may allow the differentiation of FRA from purely psychogenic amnesia in this patient whose neurological and neuropsychological assessment was otherwise unremarkable. We are aware of the potential risk that variability between individuals (irrespective of specific pathological symptoms) may confound single-case versus group comparisons. However, ‘perfect matching’ with regard to all cognitive factors is impossible due to the large number of neuro-cognitive domains. We believe that using a control group that is carefully matched not only for age and gender but also for educational background and current status attenuates the potential of contingent findings. Therefore we propose that VBM-based approaches have considerable potential in patients with neuropsychological disorders lacking a clear lesion in clinical MRI and who lack a previous history of neurological, psychiatric or psychogenic disorders. Due to the potential confound induced by the comparison between a single case with a group of controls, a correspondence between the affected areas and areas which have been attributed to the impaired neuro-cognitive function are necessary to corroborate the assumed causal relationship.

It may be argued, that (sub)clinical seizure-activity lead to a functional disruption of neocortical networks involved in storage or retrieval of remote memories [3]. However, antiepileptic treatment clearly improved déjà-vues and yielded normal EEG-findings while the FRA persisted. Since seizures were not witnessed, unrecognized head trauma might have caused FRA in our patient, as previously reported [7]. Yet, MR-imaging including T2* did not show any signs of a structural lesion. Thus, the case fulfils all criteria of a “functional retrograde amnesia” [5]. It is still controversial, whether this concept is of organic or/and psychogenic etiology [6]. In our patient, neither a detailed psychiatric interview nor psychometric testing revealed any predisposing factors for a psychogenic amnesia [8]. On the contrary, VBM-analysis corroborates that FRA was not purely a psychogenic memory loss, as it disclosed grey matter abnormalities in left temporopolar and right posterior parahippocampal/lingual cortex.

It is controversial which brain structures underlie the retrieval of past memories. In a meta-analysis on neuroimaging studies of autobiographic memory (AM) [25], a network of the regions associated with AM processing was identified and classified into “core regions”, “secondary regions” and “regions that were infrequently activated across studies”. Here, core regions included the medial and ventrolateral prefrontal cortices, medial and lateral temporal cortices, temporoparietal junction, retrosplenial/posterior cingulate cortex and the cerebellum [25]. With respect to our findings, the left temporal pole is considered to be a “secondary region” of autobiographical memory processing according to this classification. The second region found in our study, the posterior parahippocampal/lingual cortex most closely corresponds to a broader region in the metaanalysis, the right medial temporal lobe. This region is classified as core region according to this metaanalysis and not further subdivided. In a second meta-analysis on neuroimaging studies of AM both left temporal pole and right parahippocampal cortex are found to be regions that show significant concordance across neuroimaging studies in healthy subjects [26]. In this study, peak coordinates of all clusters are reported which allowed us to compare the distance between the peak coordinate in our study (after conversion from MNI into Talairach coordinates [27]) with the peak coordinate reported in this meta-analysis. The largest distance between both peak coordinates of the temporal pole cluster amounts to 13 mm (along the z-axis), while the largest distance between both peak coordinates in the right parahippocampal cluster amounts to 22 mm. Thus the abnormalities found in our patient project to regions relevant for AM also according to meta-analyses in healthy volunteers. The rather large distance between the peak within the clusters and the findings in our patient may stem from: (i) the analytical difference between peak localization and cluster distribution, (ii) the variance between the different operationalizations of AM, and (iii) a potential interindividual variance. Furthermore it seems relevant to compare our findings in a single patient to previous reports in other patients with similar amnestic syndromes [2], [5], [6], [7], [8], [28], [29], [30]. In this vein, previous studies reported that lesions of the temporopolar cortex might result in FRA [2], [28]. A meta-analysis of published cases of FRA suggests, that damage to the anterior temporal lobe results in pronounced impairment of episodic but preserved semantic memory [29]. This corresponds to the pattern we found in our patient.

The majority of reported cases with retrograde memory deficits show multiple as opposed to isolated lesions [30], [4]. Similarly our patient also showed VBM-based grey matter abnormalities in the border of posterior parahippocampal (PHC) and lingual cortex. PHC is part of the medial temporal lobe and contributes to fundamental functions sustaining retrograde memory [30], [4]. It has been suggested, that lesions confined to the hippocampus proper result in a temporally graded retrograde amnesia, while lesions involving adjacent areas, like PHC, cause severe, temporally extensive and ungraded amnesia which converges with the findings in our patient [30]. Moreover, PHC is involved in familiarity judgements of memories. Interestingly, temporal lobe epilepsy patients have been reported to show interictal hypometabolism in this region associated with déjà-vues, a frequent symptom prior to anticonvulsant therapy in our patient [31]. It may be argued, that the morphological alterations in our patient are unrelated to the memory deficit and represent the mere epiphenomenon of a cryptogenic temporal lobe epilepsy (CTLE). Indeed a recent VBM-study did report abnormalities in CTLE patients [32], however, therapy refractory patients with a long history and high seizure frequency were enrolled (∼35 seizures/year; epilepsy duration: ∼24 years). On the contrary our patient had suffered only one seizure rendering tissue damage due to repetitive and sustained seizure activity unlikely. Hence we consider the subtle parahippocampal/lingual and temporopolar alterations disclosed by VBM-analysis to represent structural correlates of the deficit in the here reported FRA-case. It cannot be entirely excluded that additional covert damage to other temporal lobe regions that was not detected by our analysis might contribute to the patient's deficit. Nevertheless, our findings point out that very subtle structural abnormalities in critical structures of the memory network might result in pronounced deficits of the autobiographical memory. The fact that we found abnormalities in two different structures on both hemispheres, does not allow us to infer specific contributions of each structure or its laterality to the memory deficit in our patient. Nevertheless our results agree with the notion, that combined lesions of MTL and neocortical structures can lead to FRA [30], [4]. Beyond the implications for the diagnosis of FRA our results advocate the use of VBM in conditions that do not show abnormalities in clinical neuroradiological assessment.
